# Supplementary material for: Beyond Multiparametric MRI and towards Radiomics to Detect Prostate Cancer: A Machine Learning Model to Predict Clinically Significant Lesions
Source: Cancers (Basel). 2022 Dec 14;14(24):6156. doi: 10.3390/cancers14246156 (PMC9776977; doi:10.3390/cancers14246156)

Supplementary Files

Table S1. Scan parameters of DWI sequences

Parameter	DWI
**Pulse sequence**	Echoplanar imaging (EPI)
**Orientation**	Axial
**Repetition time [ms]**	8000
**Echo time [ms]**	84
**Flip angle [degree]**	90
**Matrix size**	128 × 128
**Pixel Spacing [mm]**	0.78
**Slice thickness [mm]**	3
**Slice spacing [mm]**	0.3
**b-value [s/mm^2]**	0/50/1000

Supplementary Materials and Methods

Comparison between original and augmented predictive models of GG≥3

A preliminary predictive model was developed on the original study population made by 117 PCa lesions, split between 61 GG<3 and 56 GG≥3 using the 4-dim feature vector arising from the feature selection procedure. Then, the original study population has been divided into training and test sets exploiting the Support Vector Machine (SVM) margin rule described in the "*Training and test of the predictive model of GG≥3*" section of the main manuscript. Accordingly, the training set was made by 87 lesions (45 GG<3 and 42 GG≥3), whilst the test set was of 30 lesions (16 GG<3 and 14 GG≥3). In addition, the training set was randomly split into three folds to perform Cross Validation (CV), so that each fold contained 29 lesions, that is 15 GG<3 and 14 GG≥3. This predictive model using the original study population relies on the same procedure described in the main manuscript for the model exploiting the augmented features. The Supplementary Table 2 reports its performance on the training and test sets, separately. Then, to prove the statistical representativeness of the augmented features, the performance of the two models (i.e., the original and the augmented one) has been compared. In particular, the Receiver Operating Characteristic (ROC) curves have been built for both models and linearly interpolated between 0 and 1 (i.e., specificity/sensitivity ranges), using a step of 0.1. The equivalence between the ROC curves of training and test sets, separately, has been tested using a Wilcoxon rank-sum test ($p<0.001$). Supplementary Figure 2 shows the original and interpolated ROC curves for the original study population (i.e., Initial Dataset, ID) and the augmented one (i.e., Oversampled Dataset, OD) for training (a) and test sets (b) respectively. The ROC curves of the two models resulted equivalent in both training and test, with $p=0.57$ for training and $p=0.003$ for test.

Table S2. Performance of the predictive model in training and test sets referring to the original study population made by 117 PCa lesions

Metric	Training (87 samples)	Test (30 samples)
**AUC**	0.87	0.85
**SN**	86%	86%
**SP**	82%	81%
**I**	0.68	0.67
**PPV**	82%	80%
**NPV**	86%	87%
**FP**	8	3
**FN**	6	2

Figure S1. Comparison of Receiver Operating Characteristic (ROC) curves achieved by the predictive models coming coming from the original (i.e., Initial Dataset, ID) and augmented (i.e., Oversampled Dataset, OD) study population for training (a) and test (b) sets. In particular, continuous lines are the linearly interpolated curves of the sample ROC curves shown by the dotted lines.

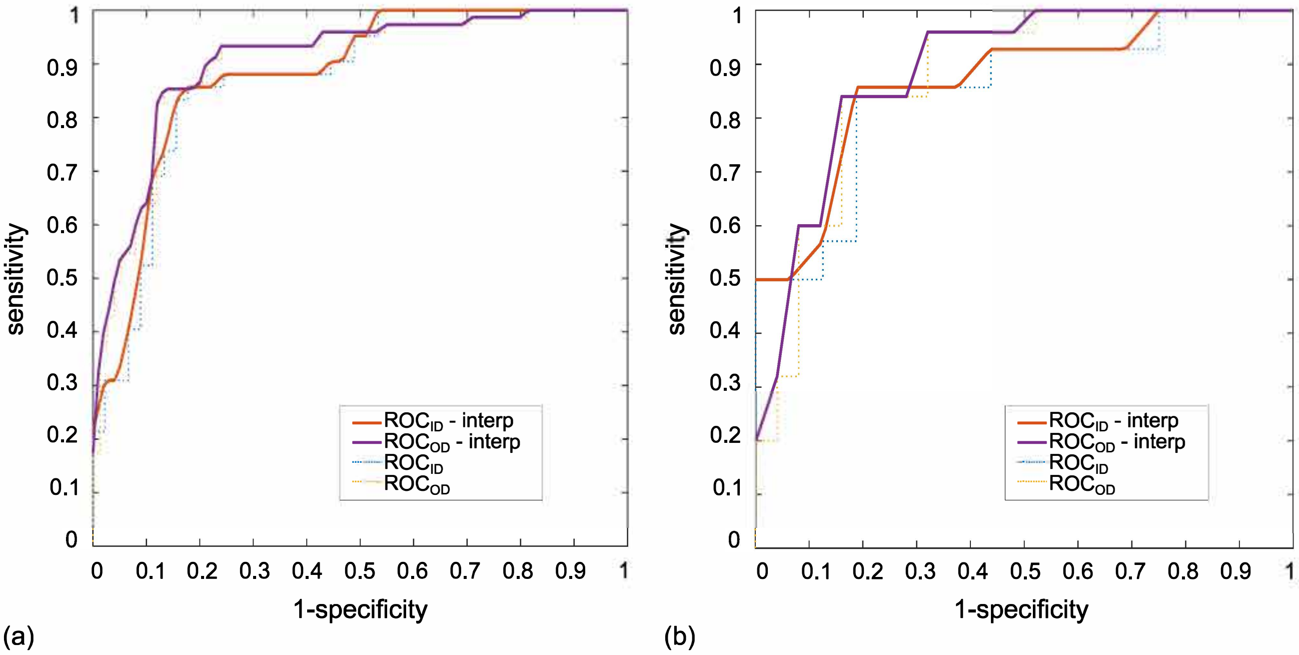

Supplement: Supplementary file 1 [file cancers-14-06156-s001.zip › cancers-2053183-supplementary.pdf]
